# Spatial compartmentalization shapes distinct germinal center states during respiratory viral infection

**DOI:** 10.3389/fimmu.2026.1930085

**Published:** 2026-09-30

**Authors:** Dong-Yeon Kim, YoungMin Woo, Taehwan Oh, Green Kim, Seung Ho Baek, Eun-Ha Hwang, Gukhui Min, Miji Kim, You Jung An, Yujin Kim, Kyong-Cheol Ko, Jung Joo Hong

**Affiliations:** 1 National Primate Research Centre, Korea Research Institute of Bioscience and Biotechnology (KRIBB), Cheongju, Chungcheongbuk, Republic of Korea; 2 KRIBB School of Bioscience, Korea University of Science & Technology (UST), Daejeon, Republic of Korea; 3 Department of Microbiology, College of Medicine, Dankook University, Cheonan-si, Chungcheongnam-do, Republic of Korea; 4 Korea Preclinical Evaluation Center, KRIBB, Daejeon, Republic of Korea

**Keywords:** germinal centers, mediastinal lymph nodes, medulla, nonhuman primate, SARS-CoV-2

## Abstract

**Background:**

Germinal centers (GCs) typically arise within the lymph node cortex, where follicular dendritic cell (FDC) networks and follicular helper T cells coordinate B-cell expansion, affinity maturation, and durable immunity. Whether proliferating B-cell aggregates in the medulla engage bona fide GC programs or instead represent a distinct GC-like differentiation state remains unresolved.

**Methods:**

In this study, using mediastinal lymph nodes from SARS-CoV-2-infected cynomolgus macaques, we combined immunofluorescence with Xenium spatial transcriptomics to identify LYVE1+ sinus-associated medullary GC structures (mGCs) alongside conventional cortical GCs (cGCs).

**Results:**

mGCs shared core GC-associated modules with cGCs but showed niche-divergent features, including increased expression of PRDM1, MZB1, and VWF and decreased expression of IRF8, CD27, and CD86. mGCs retained organized follicular architecture but showed lower CD4+ T and GC B cell densities, greater plasma cell representation, and enrichment of pDCs, NK cells, and stromal populations. Compared with cGCs, mGCs lacked polarized CD35+ FDC and CD3+ T cell network topology, exhibited reduced CXCL12 availability, and showed a higher IgM/IgG ratio. During early reinfection, medullary secondary follicles contracted sharply and were associated with higher Foxp3+ cell density.

**Conclusion:**

These findings support a model in which lymph node microanatomy imprints a distinct mGC state, diversifying humoral immune architecture during respiratory viral infection.

## 1 Introduction

Germinal centers (GCs) are specialized microanatomical structures within secondary lymphoid organs that serve as the primary sites of B cell clonal expansion, somatic hypermutation, and antibody affinity maturation during T cell-dependent immune responses (1). Within GCs, B cells undergo iterative cycles of mutation in the dark zone (DZ) and affinity-based selection in the light zone (LZ), a process that progressively enriches for high-affinity clones and gives rise to long-lived plasma cells and memory B cells (1–3). The selection of GC B cells in the LZ depends critically on interactions with follicular dendritic cells (FDCs) and follicular helper T (Tfh) cells, which together provide the survival and proliferative signals that determine clonal fate (4, 5).

GCs are classically described as arising within the follicular cortex of lymph nodes, where a dense network of FDCs, abundant B cells, and T–B border proximity collectively support GC initiation, DZ–LZ polarity, and sustained reactions (1, 6–8). In contrast, the lymph node medulla has long been regarded as a region associated primarily with lymphocyte egress, antibody secretion, and the residence of differentiated plasma cells, lacking the stromal organization thought necessary for GC formation (6, 9). However, recent studies have reported BCL6^+^ GC-like proliferative structures in the medullary regions of inflamed human lymph nodes and follicle-like B cell nodules in the medulla of murine mucosa-draining lymph nodes (10, 11), raising the possibility that productive GC-like activity is not strictly confined to the cortex. Whether these medullary structures engage canonical GC transcriptional programs or represent a distinct form of B cell organization has not been resolved.

Defining the identity and properties of medullary GC-like structures has been limited by the technical challenge of resolving tissue architecture and gene expression within intact sections. Conventional immunofluorescence identifies proliferating B cell clusters but cannot capture the underlying molecular programs that confer bona fide GC identity. Recent advances in imaging-based spatial transcriptomics now enable gene expression profiling directly within native tissue microenvironments at single-cell resolution. In our previous work applying Xenium-based spatial transcriptomics to lung-draining medLNs from SARS-CoV-2-infected cynomolgus macaques, we observed proliferating B cell aggregates adjacent to LYVE1^+^ medullary sinuses—a location not traditionally associated with GC formation—motivating a systematic comparison with conventional cortical GCs (12).

Here, we systematically characterized these medullary GC-like structures (mGCs) and compared them with conventional cortical GCs (cGCs) within the same lymph node sections. Using immunofluorescence and Xenium-based spatial transcriptomics, we show that mGCs retain core GC-associated transcriptional features while exhibiting niche-conditioned differences in architecture, cellular composition, chemokine landscape, isotype balance, and persistence after secondary challenge. Together, these findings support the existence of a spatially distinct GC state with distinct molecular features within the lymph node, rather than simply reflecting displacement of canonical cortical GCs, and highlight tissue compartment identity as a determinant of humoral immune organization.

## 2 Materials and methods

### 2.1 Cell culture and virus propagation

Vero cells (CCL-81; ATCC) were cultured in Dulbecco’s Modified Eagle Medium (DMEM; Welgene Inc.) supplemented with 10% fetal bovine serum (FBS) and 1% penicillin–streptomycin. The SARS-CoV-2 variants used in this study (NCCP no. 43326, S clade [A, 19B], NCCP no. 43390, GK clade [B.1.617.2 lineage], NCCP no. 43408, GRA clade [B.1.1.529 lineage]) were obtained from the National Culture Collection of Pathogens (Cheongju, Korea). Viral stocks were propagated in Vero cells, and infectious titers were determined using the Reed–Muench method (13). All experiments involving infectious SARS-CoV-2 were performed in a Class II biosafety cabinet under animal biosafety level 3 (ABSL-3) containment at the Korean National Primate Research Center, Korea Research Institute of Bioscience and Biotechnology (KRIBB), under an approved permit (KRIBB-IBC-20200206).

### 2.2 Animals and study design

A total of 28 cynomolgus macaques (*Macaca fascicularis*; males and females, 5–7 years of age) with no prior exposure to SARS-CoV-2 were selected based on physical examination and hematologic screening performed by the institutional veterinarians. Animals were housed individually in indoor cages under controlled temperature, humidity, and light cycles within the ABSL-3 Biocontainment Laboratory at the Korean National Primate Research Center, KRIBB. All animal procedures were conducted in accordance with institutional guidelines and approved by the KRIBB Institutional Animal Care and Use Committee (KRIBB-AEC-20064, 21289, and 22230).

All animals were anesthetized with ketamine hydrochloride (10 mg/kg, IM) for blood collection, virus inoculation, and other experimental procedures. For infection, 21 macaques were inoculated with a total volume of 10.5 mL of SARS-CoV-2 Wuhan, Delta, and Omicron variants (2.1 × 10^6^ TCID_50_/mL) administered through multiple mucosal routes: intratracheal (4 mL), intranasal (1 mL), oral (5 mL), and conjunctival (0.5 mL), following established protocols (14, 15). The animals necropsied at 31 dpi were rechallenged with the SARS-CoV-2 Omicron variant at 28 dpi and euthanized 3 days later for tissue collection and analysis. The remaining 7 macaques served as uninfected donors for spleen-based ex vivo experiments; of these, 3 animals were also used as naïve controls for immunofluorescence analysis of the medLNs.

### 2.3 Tissue preparation

At the study endpoint, animals were anesthetized with ketamine hydrochloride (10 mg/kg, IM) and euthanized with sodium pentobarbital (100 mg/kg, IV) in accordance with the AVMA Guidelines for the Euthanasia of Animals (2020 Edition). MedLNs were harvested at necropsy from animals at naïve, 3, 21, and 31 dpi. To prepare single-cell suspensions, spleen tissue fragments were placed in MACS C Tubes (Miltenyi Biotec) containing 5 mL of Roswell Park Memorial Institute (RPMI)-1640 supplemented with 10% FBS and 1% penicillin–streptomycin and homogenized using a gentleMACS Dissociator (Miltenyi Biotec). For histological and imaging analyses, tissues were fixed in 4% paraformaldehyde (Biosolution) at 4 °C for 24–48 h, dehydrated through graded ethanol and xylene, and embedded in paraffin. Serial sections (3–5 μm thick) were prepared for immunofluorescence staining and Xenium *in situ* spatial transcriptomic analyses.

### 2.4 Immunofluorescence staining

FFPE medLN sections were deparaffinized, rehydrated, and subjected to heat-mediated antigen retrieval using Diva Decloaker (Biocare) in a Decloaking Chamber. The sections were permeabilized with 0.1% Triton X-100 in PBS and blocked with 4% donkey serum. The primary antibodies were diluted in blocking buffer and incubated overnight at 4 °C or for 1 h at room temperature. The following primary antibodies were used: CD3 (CD3-12; Bio-Rad; 1:200), CD35 (E11; Abcam; 1:400), IgD (SouthernBiotech; 1:200), IgM (SA-DA4; SouthernBiotech; 1:100), IgG (SouthernBiotech; 1:100), KI-67 (B56; BD; 1:200), BCL6 (PG-B6p; Dako; 1:100), and CXCL12 (SDF-1; Abcam; 1:100). After washing with TBS and TBST, Alexa Fluor–conjugated donkey secondary antibodies (Jackson ImmunoResearch; 1:2000) were applied for 30 min at room temperature. The slides were mounted with an anti-fade medium (Abcam) and imaged using an Olympus IX83 fluorescence microscope (Olympus).

### 2.5 Imaging-based spatial transcriptomics

*In situ* RNA expression analysis was performed using the Xenium platform (10x Genomics) according to the manufacturer’s instructions and previously established workflows (12). A 377-probe Xenium Human Multi-Cancer Panel was used, and probe performance was confirmed using macaque reference datasets (16). Transcript decoding and DAPI-guided segmentation were performed using Xenium Analyzer (v1.1.0.2). Matrices and spatial coordinates were analyzed using Seurat (v4.9.9.9050) with SCTransform normalization and Harmony-based batch correction. Clustering was based on the first 17 principal components (resolution = 0.5), and cell type annotation was guided by marker genes identified in our previously established single-cell atlas of macaque medLNs. The same annotation framework was applied to ensure consistency across the datasets.

### 2.6 Spatial compartment assignment and ROI strategies

GC-containing follicles were identified as IgD^lo^ regions containing MKI67^hi^ proliferative clusters and classified into cGC or mGC compartments according to their spatial relationship with LYVE1^+^ medullary sinus structures. Follicles directly associated with LYVE1^+^ sinus regions were defined as mGCs, whereas anatomically separated cortical follicles were classified as cGCs, and ambiguously positioned follicles were excluded to avoid classification bias. For Xenium spatial transcriptomic analysis, ROIs encompassed entire follicular structures, including both GC core and mantle zone to preserve anatomical context and enable computational cell-type separation at single-cell resolution. In contrast, immunofluorescence-based protein quantification was restricted to the IgD^lo^ GC core to avoid signal dilution from surrounding mantle zone naïve B cells and enhance detection of compartment-specific differences in GC-associated proteins. Together, these complementary ROI strategies enabled integrated transcriptional and protein-level characterization of cGC and mGC compartments.

For spatial quadrant analysis of immunofluorescence images, we adapted the approach described by Pikor et al. (17), in which GCs are divided into four equivalent quadrants according to anatomical orientation. For cGCs, Q1 was oriented toward the subcapsular sinus and Q4 toward the T-cell zone. For mGCs, the same framework was adapted to the medullary anatomy, with Q1 oriented toward the LYVE1^+^ medullary sinus interface and Q4 toward the T-cell zone. Quadrant boundaries were manually assigned, but their orientation was standardized using these predefined anatomical landmarks. GC classification, follicle counting, quadrant assignment, and immunofluorescence quantification were performed in a blinded manner and reviewed by a veterinarian.

### 2.7 GSEA of gene ontology pathways

The differential expression between cGCs and mGCs was calculated using Seurat (FindMarkers, Wilcoxon rank-sum test; adjusted *p <* 0.05). Genes were ranked by log2 fold-change and analyzed using Gene Set Enrichment Analysis (GSEA, v4.3.3) with the C5 GO gene set collection from Molecular Signatures Database (MSigDB; v2025.1). Gene symbols were standardized using the Human_Gene_Symbol_with_Remapping_MSigDB.v2025.1.Hs annotation resource. Statistical significance for enrichment was computed using 1,000 permutations.

### 2.8 Spatial cell-location mapping and Ripley’s multitype K function analysis

Cell-location maps were generated from Xenium-derived cell centroid coordinates. GC ROIs were manually defined for cGCs and mGCs, and cell-level ROI tables were merged with Xenium cell centroid coordinates to obtain the x and y positions of individual cells within each ROI. PDCD1^+^CD4^+^ T cells were defined as CD4^+^ T cells containing one or more PDCD1 transcripts.

For spatial point-pattern analysis, GC B cells and PDCD1^+^CD4^+^ T cells within each ROI were converted into marked point-pattern objects in R (version 4.5.1). Multitype K function analysis between GC B cells and PDCD1^+^CD4^+^ T cells was performed using the Kcross function in the spatstat package (version 3.4-3) (18). The observed K function, K_obs_(r), was calculated across spatial distances up to 150 μm and compared with the theoretical K function expected under a homogeneous Poisson process, K_pois_(r). ROI-level K functions were summarized separately for cGCs and mGCs by calculating the pointwise mean with pointwise 95% confidence interval (gray).

### 2.9 Cell stimulation

Normal splenocytes (5 × 10^4^ cells/well) were plated in 96-well plates in RPMI 1640 medium, supplemented with 10% FBS and 1% penicillin–streptomycin. Cells were stimulated under two conditions: (1) CpG (1 μg/mL) alone and (2) CpG (1 μg/mL) combined with CXCL12 (25 ng/mL). The plates were incubated at 37 °C and 5% CO_2_ for 4 days.

### 2.10 ELISpot

ELISpot assays were performed as previously described (15). Briefly, PVDF 96-well plates (Millipore) were coated with goat anti-monkey IgG/IgM (10 µg/mL; Rockland) overnight at 4 °C. Stimulated splenocytes were incubated for 4 h in CTL-Test Medium (Cellular Technology Ltd.). IgG- and IgM-secreting cells were detected using FITC-conjugated anti-IgG and biotin-conjugated anti-IgM, followed by FITC-HRP and streptavidin-AP. Spots were developed and counted using an ImmunoSpot S6 Universal Analyzer (CTL), and the results were expressed as spot-forming cells per 1,000,000 cells.

### 2.11 Statistical analysis

Statistical analyses were performed using GraphPad Prism (v10). Depending on the experimental design, either an unpaired Mann–Whitney test, a paired Wilcoxon test, or a repeated-measures one-way ANOVA with appropriate multiple comparison corrections was applied. The specific statistical tests used for each dataset are indicated in the corresponding figure legend. Statistical significance was set at *p <* 0.05.

## 3 Results

### 3.1 SARS-CoV-2 induces orthotopic and ectopic GC structures in lung-draining medLNs

To map the spatiotemporal emergence of GC reactions in lung-draining lymph nodes after SARS-CoV-2 infection, we analyzed medLNs from cynomolgus macaques at 0-, 3-, and 21-days post-Omicron (B.1.1.529 lineage) infection (**Figure 1A**). We operationally defined putative GC structures as KI67^hi^, proliferative clusters within IgD^lo^ follicles, consistent with BCL6 expression patterns (**Supplementary Figure 1**). KI67^hi^ clusters were not detected in the uninfected controls (**Figure 1B**). By 3 dpi, KI67^hi^ clusters appeared predominantly at the orthotopic sites, with only rare clusters outside the cortex (**Figure 1C**). By 21 dpi, medLNs contained abundant orthotopic cortical GCs and numerous non-cortical (ectopic) KI67^hi^ clusters, reaching counts comparable to cortical GCs within the same sections (**Figure 1D**). Similar ectopic patterns were observed in medLN from macaques infected with the Wuhan strain and Delta variant (**Supplementary Figure 2**), indicating that ectopic GC-like structures are not restricted to Omicron infection. These observations motivated a molecular and spatial comparison of cortical and non-cortical GC structures using imaging-based spatial transcriptomics.

**Figure 1:**
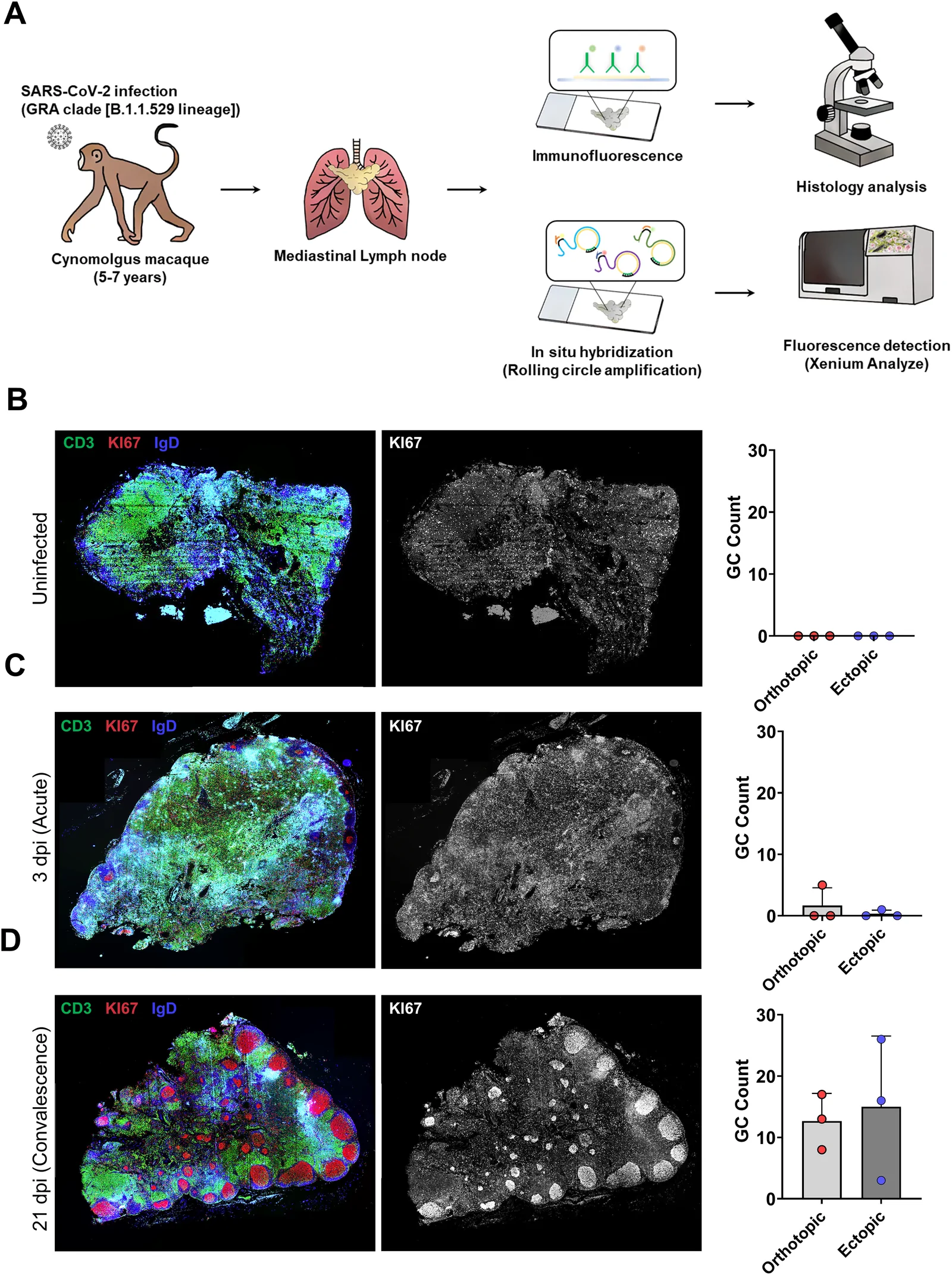
Germinal center formation in medLNs following SARS-CoV-2 Omicron infection. **(A)** Schematic overview of the experimental design. **(B)** Representative immunofluorescence images of uninfected medLNs and quantification of GC counts per medLN section. **(C)** Representative immunofluorescence images of medLNs at 3 dpi and quantification of GC counts per medLN section. **(D)** Representative immunofluorescence images of medLNs at 21 dpi and quantification of GC counts per medLN section. CD3 (green), KI67 (red), and IgD (blue). n = 3 animals per group; one medLN section per animal was analyzed.

### 3.2 LYVE1^+^-anchored mGCs share core GC-associated transcriptional programs and niche-divergent programs

Building on the abundant non-cortical GC-like proliferative clusters observed in infected medLNs (**Figure 1**), we applied imaging-based spatial transcriptomics (Xenium) to define their anatomical anchoring and molecular state *in situ*. A total of 377 targets from the Xenium Human Multi-Cancer Panel were profiled with robust detection across samples, and the probe performance was benchmarked against macaque reference datasets. Spatial maps of MS4A1 and MKI67 overlaid with LYVE1 demonstrated that the non-cortical MKI67^hi^ clusters reproducibly localized adjacent to LYVE1^+^ medullary regions (**Figure 2A**). Based on this consistent spatial association, we hereafter refer to these structures as mGCs and distinguish them from cGCs.

**Figure 2:**
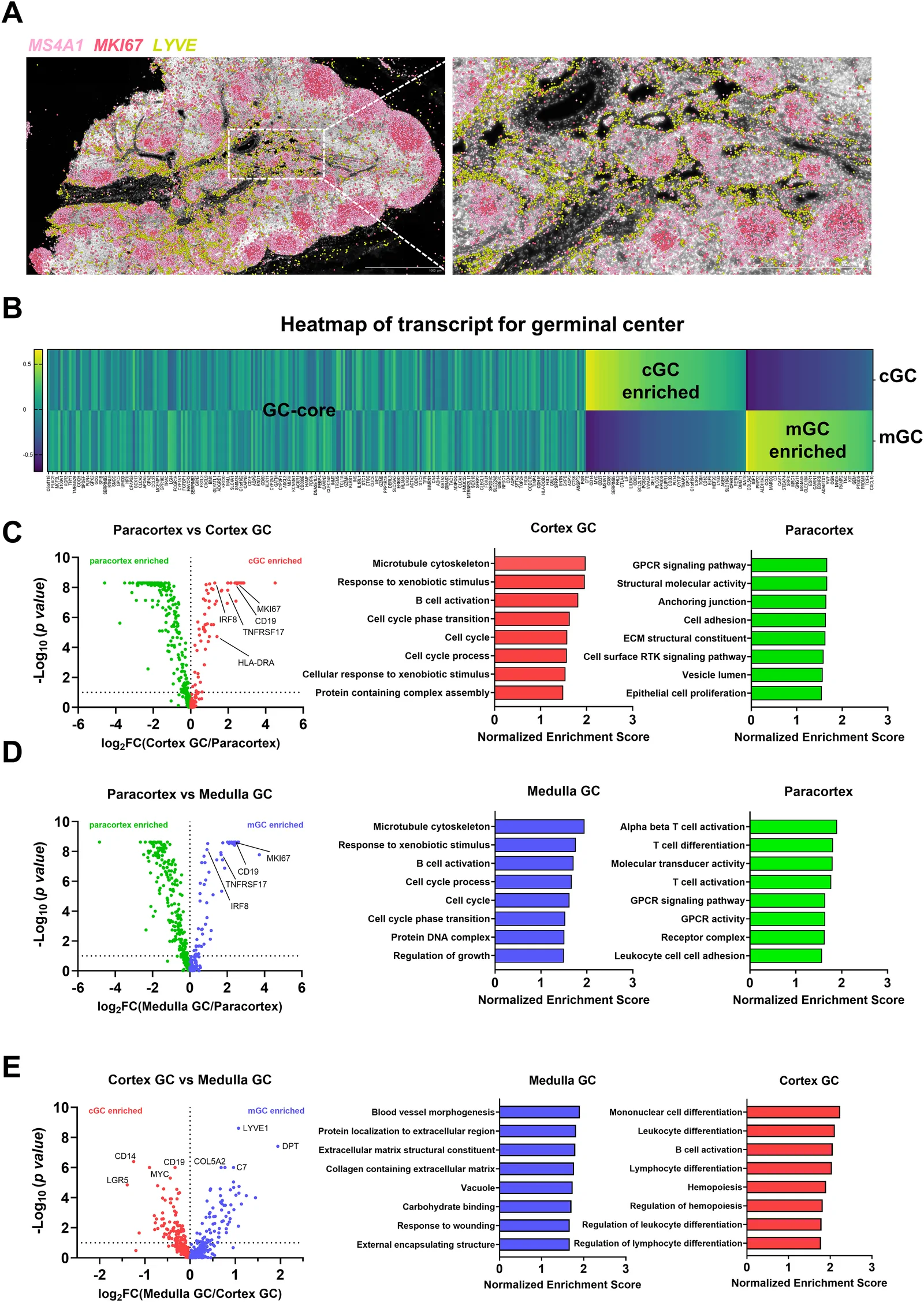
Spatial transcriptomic profiling of distinct molecular signatures in cGCs and mGCs. **(A)** Spatial transcriptomic maps of medLNs showing the distribution of MS4A1, MKI67, and LYVE1. **(B)** Heatmap of transcript expression across germinal center regions. **(C)** Differential gene expression and pathway enrichment analysis of paracortex versus cortical GCs. Left: Volcano plot of genes enriched in the cGCs (red) and paracortex regions (green). Right: GSEA-based pathway enrichment profiles for cGC- and paracortex-enriched pathways. **(D)** Differential gene expression and pathway enrichment analysis of paracortex versus medullary GCs. Left: Volcano plot of genes enriched in the mGCs (blue) and paracortex regions (green). Right: GSEA-based pathway enrichment profiles for mGC- and paracortex-enriched pathways. **(E)** Differential gene expression and pathway enrichment analysis of cortical GCs versus medullary GCs. Left: Volcano plot of genes enriched in the cGCs (red) and mGC (blue). Right: GSEA-based pathway enrichment profiles for mGC- and cGC-enriched pathways. Spatial transcriptomic analyses were performed on medLNs from n = 3 animals, with one medLN analyzed per animal. Across the three animals, a total of 28 cGC, 31 mGC, and 20 paracortex ROIs were analyzed. ROIs from each region were pooled for downstream differential gene expression and pathway enrichment analyses.

To assess the transcriptional identity of mGCs relative to cGCs and the paracortex, we first examined transcript-level expression patterns across all 377 profiled targets. cGCs and mGCs share a broadly overlapping transcriptional landscape across various assayed genes, consistent with a common GC-associated foundation (**Figure 2B**). A subset of gene clusters showed compartment-associated expression patterns, indicating that niche-dependent programs are superimposed on this shared GC framework rather than representing wholesale transcriptional divergence (**Figure 2B**).

To characterize the functional content of these shared and divergent programs, we performed differential expression analysis, benchmarking each GC compartment against the paracortex. Both cGCs and mGCs showed significant enrichment for microtubule cytoskeleton organization, response to xenobiotic stimulus, B cell activation, cell cycle phase transition, and cell cycle progression pathways relative to the paracortex (**Figures 2C, D**), supporting the annotation of medullary proliferative clusters as GC-like structures rather than nonspecific B cell aggregates. Direct transcriptional comparison between cGCs and mGCs further revealed compartment-associated signatures (**Figure 2E**). Genes relatively enriched in cGCs included B cell identity-, activation-, and selection-associated markers such as CD19 and MYC, whereas mGC-enriched genes included stromal-, vascular-, and extracellular matrix-associated factors such as LYVE1, DPT, C7, and COL5A2. Thus, mGCs share a core GC-associated program with cGC. Still, the medullary niche superimposes a distinct molecular state characterized by stromal and extracellular matrix signatures rather than intensified canonical selection programs.

### 3.3 mGCs show a plasma cell–skewed cellular composition

Given that cGCs and mGCs share core GC-associated programs but diverge in their region-level transcriptional signatures (**Figure 2**), we investigated whether these differences are reflected in cellular-scale spatial organization. Using cell annotation-based spatial profiling, we detected discrete clusters of GC B cells in both the cortex and medulla, with medullary GC B-cell clusters positioned adjacent to lymphatic endothelial structures (**Figure 3A**), consistent with the LYVE1-associated medullary niche anchoring mGCs (**Figure 2A**). To quantify the follicular organization across compartments, we classified the follicles based on the presence of GC B cells. Primary follicles lacking GC B cells were rare in both regions, whereas secondary follicles containing GC B cells were consistently detected in the medulla at densities comparable to those in the cortex (**Figure 3B**), indicating that the medulla supports organized GC-containing follicles rather than sporadic, proliferative aggregates. Representative cGCs and mGCs showed broadly comparable gross architectures (**Figure 3C**), enabling a direct comparison of their cellular compositions. Across both compartments, B-lineage cells constituted the dominant fraction of GC-associated regions, with T cells forming a smaller component and additional immune/stromal subsets present at lower proportions (**Figure 3D**). mGCs displayed significantly reduced CD4^+^ T-cell and GC B-cell densities, together with increased plasma cell density, compared with cGCs (**Figure 3E**). In addition, several non–B/T immune and stromal populations, including NK cells, pDCs, fibroblasts, and vascular/lymphatic endothelial cells, were proportionally enriched in mGCs (**Supplementary Figure 3**), consistent with a medullary microenvironment distinct from the cortical follicular niche.

**Figure 3:**
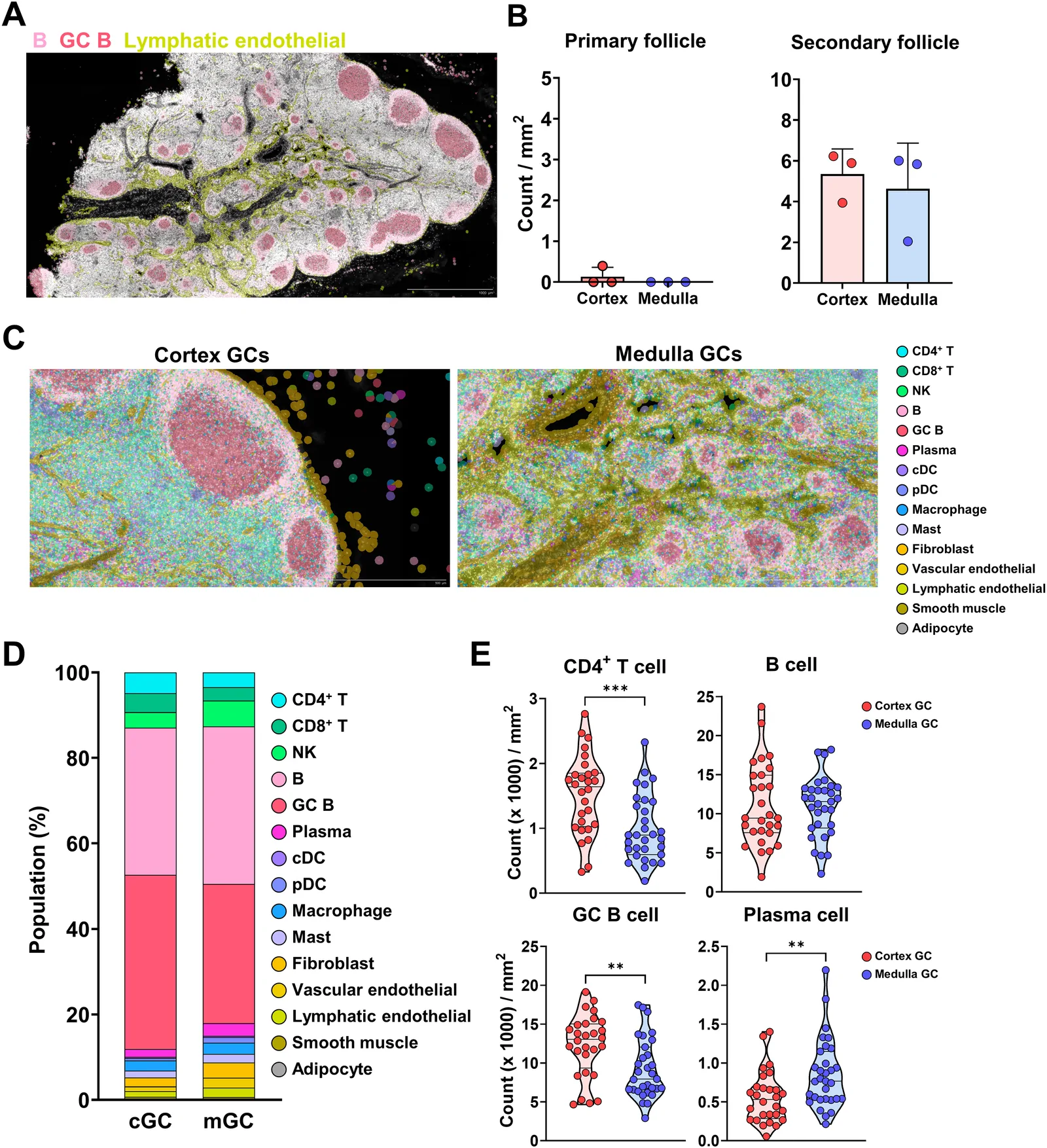
Spatial and cellular characterization of cortical and medullary germinal centers. **(A)** Spatial transcriptomic maps of medLNs showing the distribution of B cells, GC B cells, and lymphatic endothelial cells. **(B)** Quantification of primary and secondary follicles per unit area (counts per mm²) in cGCs and mGCs (n = 3 animals). **(C)** Spatial transcriptomic maps highlighting representative cGCs and mGCs, with each color indicating a distinct cell type. **(D)** Proportional composition of cell populations within cGCs and mGCs derived from the deconvolution of spatial transcriptomic data. A total of 28 cGC and 31 mGC ROIs from three animals were analyzed. **(E)** Quantitative comparison of CD4^+^ T cells, B cells, GC B cells, and plasma cell densities between cGCs and mGCs, presented as counts per mm² (28 cGC and 31 mGC ROIs from three animals). Statistical significance was assessed using the Mann–Whitney U test (**p < 0.01; ***p < 0.001).

### 3.4 mGCs lack the polarized FDC/T-cell network topology characteristic of cGCs

Having established that mGCs are anchored to a LYVE1^+^ medullary niche and exhibit distinct transcriptional and compositional features (**Figures 2**, **3**), we next tested whether a hallmark structural feature of GCs, the directional organization of stromal and T-cell networks, is maintained across anatomical sites. We performed immunofluorescence staining for CD35 (a representative light zone–associated FDC network marker) and CD3 (T cells), together with IgD, to define the follicular context (**Figure 4A**). In cGCs, CD35^+^ FDC networks were organized and displayed a strong spatial gradient, with the highest signal in Q1 and a stepwise decline toward Q4 (**Figure 4B**). A concordant quadrant-dependent decrease was also observed for CD3^+^ T cells (**Figure 4C**), indicating the coordinated directional topology of the FDC and T-cell network within cGCs. In contrast, mGCs showed CD35^+^ structures that were distributed relatively uniformly across Q1–Q4, and CD3^+^ T cells similarly lacked quadrant-dependent variations (**Figures 4D, E**). This conclusion was corroborated by line-scan analysis across the GC axis: cGCs exhibited a directional CD35 gradient from the subcapsular sinus side toward the T-B border, whereas mGCs lacked a discernible gradient along the axis defined from the LYVE1^+^ side toward the T–B border (**Supplementary Figure 4**).

**Figure 4:**
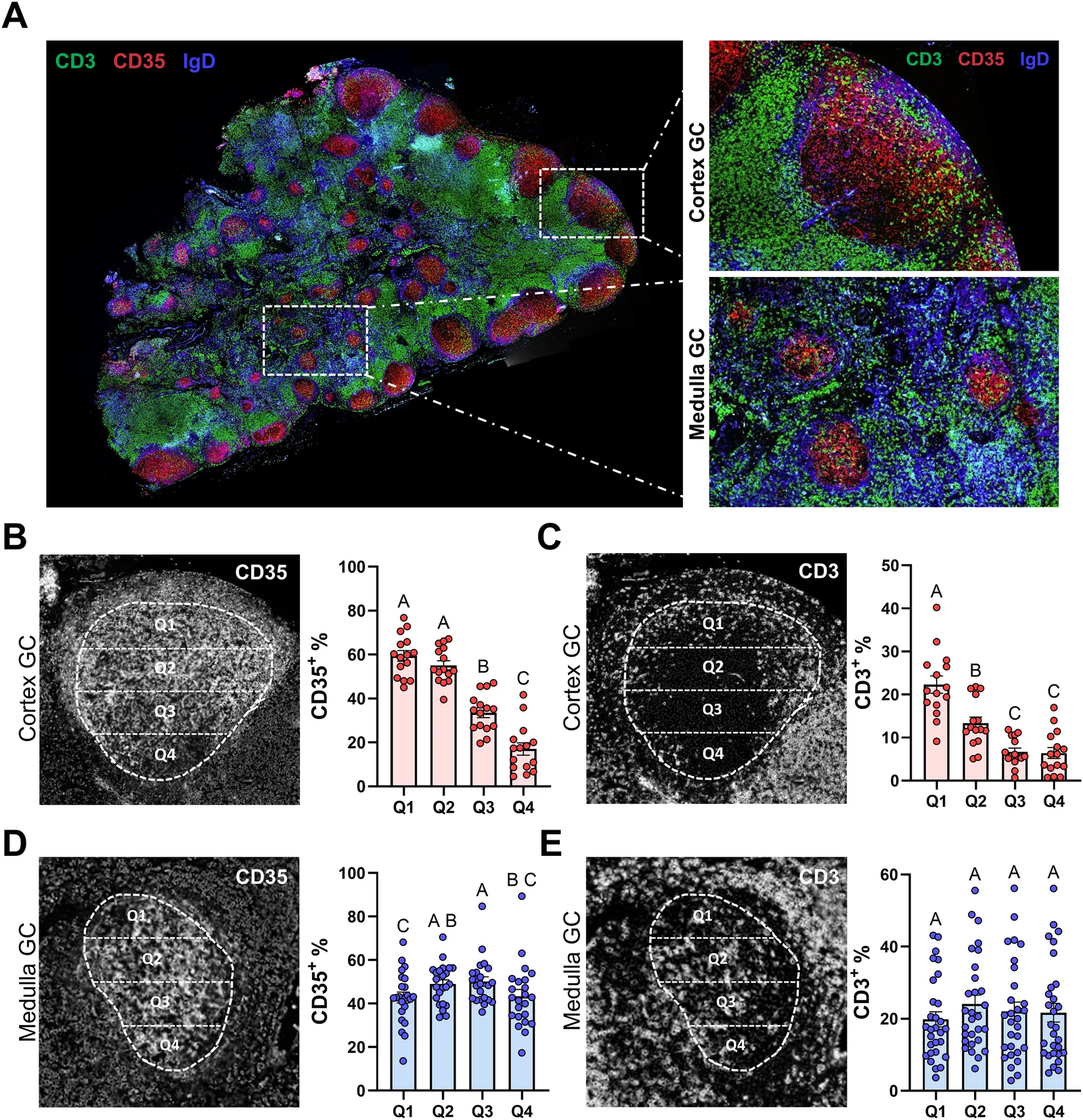
Differential spatial organization of FDC and T-cell networks in cortical and medullary germinal centers. **(A)** Representative immunofluorescence images of medLNs stained for CD3 (green), CD35 (red), and IgD (blue). Quantification of CD35^+^ FDCs **(B)** and CD3^+^ T cells **(C)** in cGCs using the same four-quadrant orientation (Q1 toward the subcapsular sinus; Q4 toward the T-cell zone). Quantification of CD35^+^ FDCs **(D)** and CD3^+^ T cells **(E)** in medullary GCs using the same four-quadrant orientation (Q1 toward LYVE1^+^ medullary sinuses; Q4 toward the T-cell zone). A total of 15 cGCs and 23 mGCs within a single medLN section were analyzed. Statistical analysis was performed using one-way ANOVA; groups labeled with different letters are significantly different (*p <* 0.05).

### 3.5 A CXCL12-limited medullary niche is associated with altered isotype balance

Having established that mGCs lack polarized FDC and T cell network topology (**Figure 4**), we asked whether this structural divergence is accompanied by altered transcriptional programs linked to B cell selection and differentiation. Because CXCL12 is a key chemokine involved in GC zonation and B cell positioning, we next examined whether the non-polarized medullary architecture was associated with altered CXCL12 availability and downstream isotype balance. We computed module scores for functionally defined gene sets across individual GC regions. The GC B cell fate program, comprising genes associated with B cell selection (IRF8, CD86, CD27, MYC, FAS, BCL2L11, CD83) and plasma cell differentiation (PRDM1, TENT5C, MZB1, FKBP11, TNFRSF17), showed clear compartment-associated gene divergence (**Figure 5A**) (4, 19–28). cGCs were relatively enriched for selection-associated genes, whereas mGCs showed preferential expression of differentiation-associated genes (**Figure 5B**). A maturation index summarizing these two modules (GC B cell selection score minus PC differentiation score) further supported the interpretation that cGCs and mGCs occupy distinct transcriptional states rather than a single uniform GC program (**Figure 5B**).

**Figure 5:**
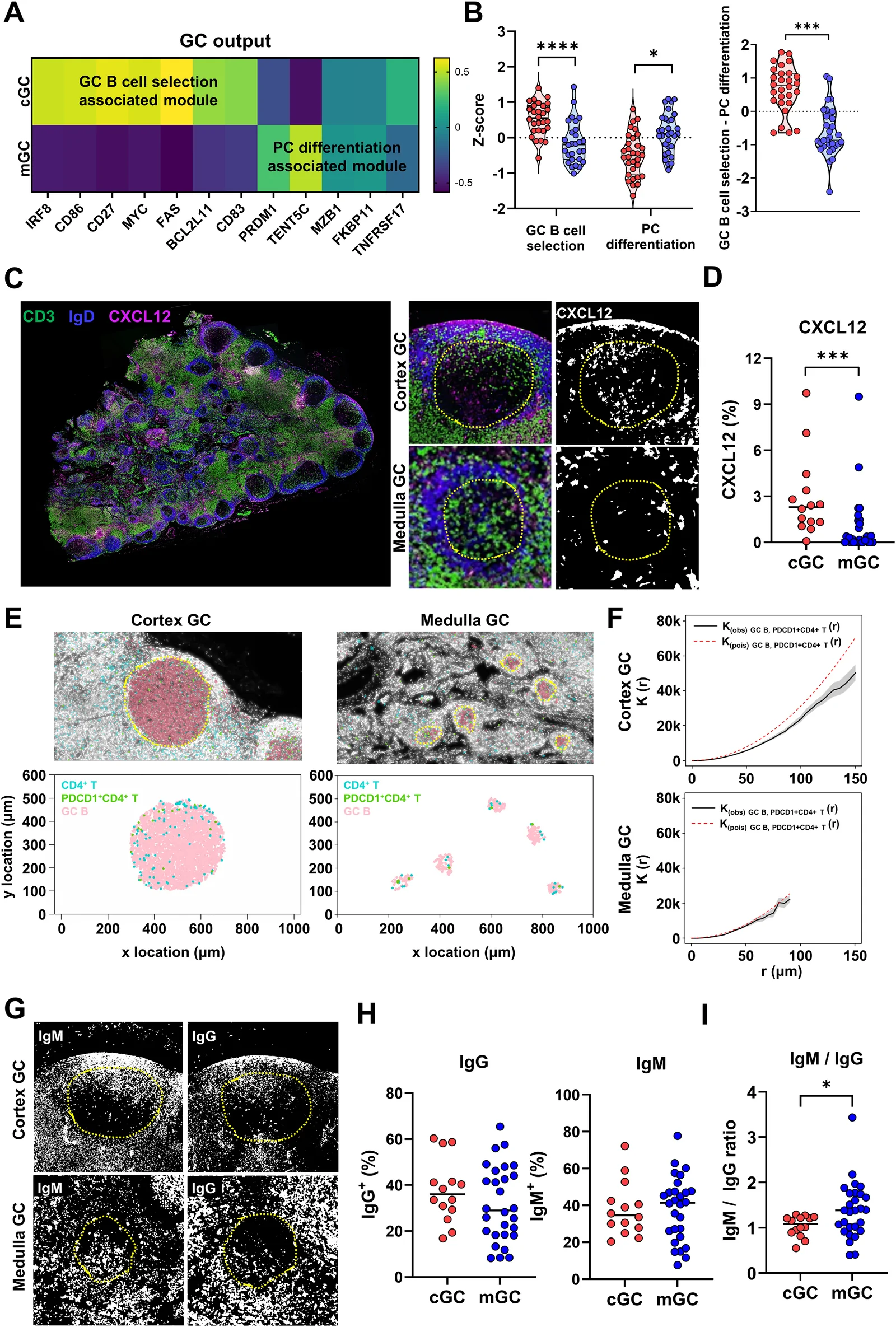
Characterization of GC output programs and CXCL12 expression in cortical and medullary germinal centers. **(A)** Heatmap of GC output program-associated genes across cGCs and mGCs. **(B)** Violin plots of GC B cell selection and plasma cell differentiation program scores in cGCs and mGCs. For **(A, B)**, 28 cGC and 31 mGC ROIs from three medLN sections from three animals were analyzed. **(C)** Representative immunofluorescence images of medLNs stained for CD3 (green), CXCL12 (magenta), and IgD (blue). **(D)** Quantification of CXCL12 expression in cGCs and mGCs. For **(C, D)**, 14 cGCs and 29 mGCs within a single medLN section were analyzed. **(E)** Representative spatial transcriptomic maps of cGCs and mGCs with analyzed GC ROIs indicated by dashed yellow outlines, and corresponding cell-location maps generated from cells within the indicated ROI; CD4^+^ T cells (mint), PDCD1^+^CD4^+^ T cells (light green), GC B cells (pink). **(F)** Summary multitype Ripley’s K function analysis of the spatial relationship between GC B cells and PDCD1^+^CD4^+^ T cells across all analyzed cGC and mGC ROIs, showing the observed K function (black) with pointwise 95% confidence interval (gray). The theoretical K function expected under a homogeneous Poisson process is shown as a red dashed line. For **(E, F)**, 28 cGC and 31 mGC ROIs from three medLN sections from three animals were analyzed. **(G)** Representative immunofluorescence images of IgM and IgG staining in cGCs and mGCs. **(H)** Quantification of IgG and IgM expression in cGCs and mGCs. **(I)** Quantification of the IgM/IgG ratio within individual GCs. For **(G–I)**, 14 cGCs and 28 mGCs within a single medLN section were analyzed. Statistical significance was assessed using the Mann–Whitney U test (*p < 0.05; ***p < 0.001; ****p < 0.0001).

We next examined whether CXCL12 availability differed between compartments. Immunofluorescence mapping revealed a prominent CXCL12 signal within cGC-associated follicular regions, whereas mGCs exhibited reduced CXCL12 staining (**Figure 5C**). Quantification confirmed significantly lower CXCL12 levels within mGC boundaries compared with cGCs (**Figure 5D**).

To further assess whether the CXCL12-limited medullary niche was associated with altered local organization of GC B cells and PDCD1^+^CD4^+^ T cells, which referred to follicular helper T cell (Tfh), we reconstructed cell-location maps from the analyzed GC ROIs in the spatial transcriptomic dataset (**Figure 5E**). Representative maps showing the locations of GC B cells, CD4^+^ T cells, and PDCD1^+^CD4^+^ T cells are presented in **Figure 5E**. To quantify the spatial relationship between GC B cells and PDCD1^+^CD4^+^ T cells, we performed a multitype Ripley’s K function analysis across cGCs and mGCs. In cGCs, the observed K function was consistently lower than the theoretical K function expected under a homogeneous Poisson process across much of the examined distance range (**Figure 5F**), indicating spatial segregation between GC B cells and PDCD1^+^CD4^+^ T cells. In contrast, the observed K function in mGCs remained close to the theoretical expectation (**Figure 5F**), indicating that the spatial segregation observed in cGCs was not evident to the same extent in mGCs. Together, these quantitative analyses indicate that the spatial relationship between GC B cells and PDCD1^+^CD4^+^ T cells differs between cGCs and mGCs, with pronounced spatial segregation in cGCs and a distribution closer to the theoretical random expectation in mGCs.

We then examined whether this altered molecular environment was associated with differences in antibody isotype distribution. Although IgM^+^ and IgG^+^ signals within individual GC regions showed inter-structure variability (**Supplementary Figure 5**; **Figures 5G, H**), the IgM/IgG ratio was significantly higher in mGCs than in cGCs (**Figure 5I**), indicating a shift in relative isotype balance in the medullary compartment rather than a uniform increase in IgM staining alone.

As a complementary ex vivo assay, we tested whether CXCL12 availability can influence class-associated antibody output in macaque lymphoid cells. Primary splenocytes from cynomolgus macaques were stimulated with CpG in the presence or absence of recombinant CXCL12, and IgM- and IgG-secreting cells were quantified by ELISpot. CXCL12 supplementation selectively increased IgG-secreting cell numbers without a corresponding change in IgM output, resulting in a reduced IgM/IgG ratio (**Supplementary Figure 6**). Because this assay does not reproduce the medLN microenvironment, we interpret it as supportive evidence that CXCL12 can influence class-specific antibody output, rather than as direct proof of mGC function.

### 3.6 mGCs-containing follicles contract during reinfection and are associated with Foxp3+ enrichment

The attenuated selection program in mGCs (**Figure 5**) raised the question of whether T cell-mediated regulatory signals also differ between compartments. GC magnitude and duration are influenced by the balance between Tfh, which promotes selection, and *Foxp3*-expressing regulatory T cells (Tregs), which can restrain GC expansion. We therefore examined whether mGCs exhibit a Treg-associated landscape that might provide context for their distinct maintenance dynamics.

Analysis of the T cell activation module revealed that most genes, including CD2, CD3D, CD4, CTLA4, IL2RA, TNFRSF9, and CD70, were relatively enriched in cGCs; FOXP3 showed consistent mGC enrichment within this module (**Figure 6A**) (29–34). PDCD1, a marker associated with GC-resident Tfh cells, showed no significant difference between compartments (**Figure 6B**) (35). These data suggest enrichment of a regulatory signature in mGCs, although FOXP3 expression alone does not establish definitive follicular regulatory T cell identity.

**Figure 6:**
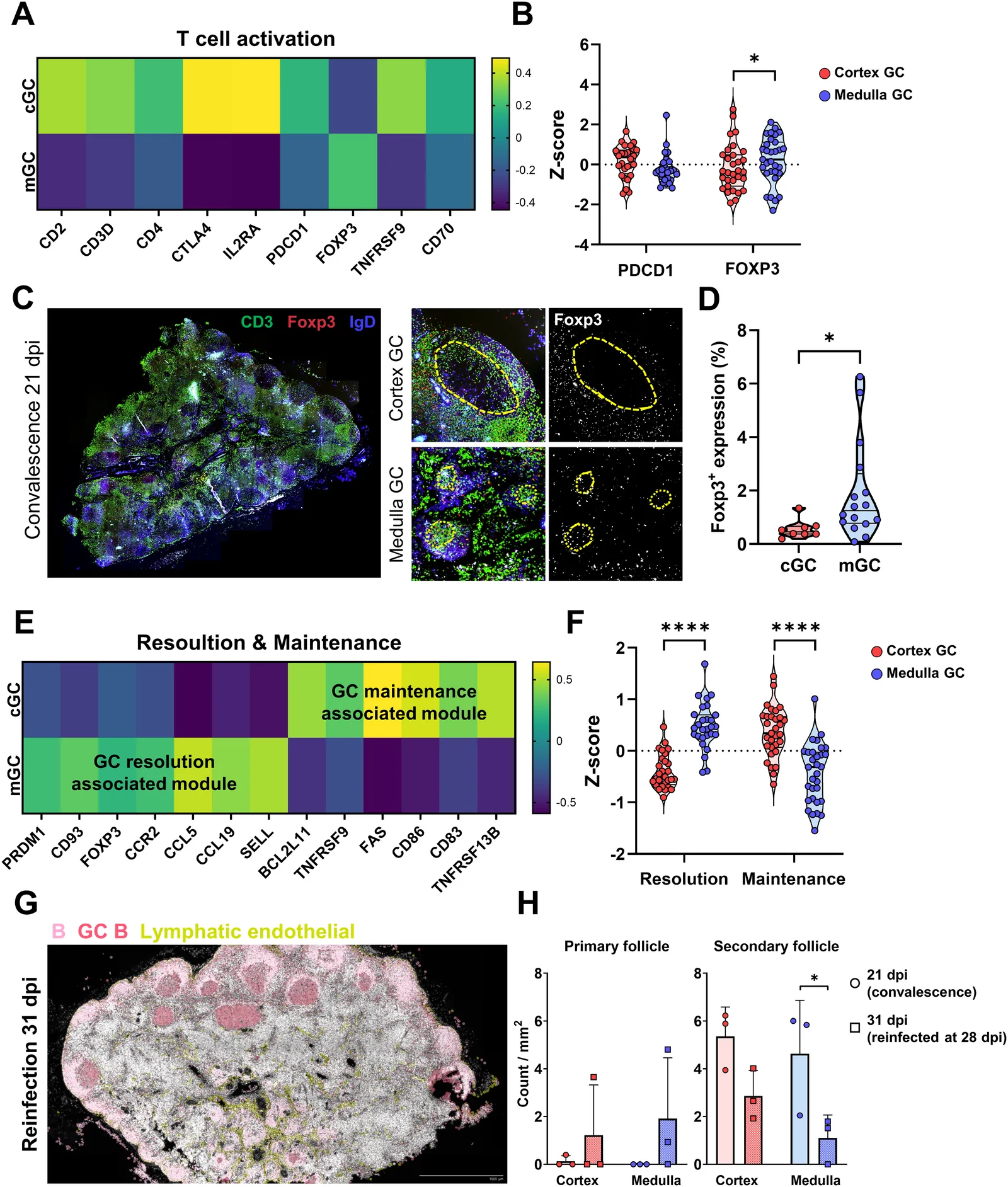
Characterization of T-cell-associated programs and GC dynamics in cortical and medullary germinal centers. **(A)** Heatmap of T cell activation-associated genes across cGCs and mGCs. **(B)** Violin plots comparing PDCD1 and FOXP3 expression scores between cGCs (red) and mGCs (blue). For **(A, B)**, 28 cGC and 31 mGC ROIs from three medLN sections from three animals were analyzed. **(C)** Representative immunofluorescence images of medLNs collected during the convalescence phase (21 dpi) stained for CD3 (green), Foxp3 (red), and IgD (blue). **(D)** Quantification of Foxp3 expression in cGCs and mGCs. For **(C, D)**, 8 cGCs and 16 mGCs within a single medLN section were analyzed. **(E)** Heatmap of contraction- and maintenance-associated genes across cGCs and mGCs. **(F)** Violin plots comparing GC resolution and GC maintenance module scores between cGCs **(red)** and mGCs (blue). For **(E, F)**, 28 cGC and 31 mGC ROIs from three medLN sections from three animals were analyzed. **(G)** Representative immunofluorescence image of medLNs collected following reinfection at 28 dpi and analyzed at 31 dpi, stained for B cells (pink), GC B cells (red), and lymphatic endothelial cells (yellow). **(H)** Quantification of primary and secondary follicles in cortical and medullary regions during the convalescence phase (21 dpi) and following reinfection at 31 dpi (n = 3 animals per group). Statistical significance was assessed using Mann–Whitney U test (*p < 0.05; ****p < 0.0001).

To validate FOXP3 enrichment at the protein level, we stained for Foxp3 at 21 dpi. Foxp3^+^ cells were visually more abundant within mGC boundaries than in cGCs within the same section (**Figure 6C**), and quantification confirmed a significantly higher Foxp3^+^ density in mGCs (**Figure 6D**).

Because FOXP3^+^ Tregs can influence GC magnitude and duration, the higher Foxp3^+^ cell density in mGCs suggested a regulatory context that could be associated with reduced persistence (36, 37). We therefore assessed whether this cellular feature was accompanied by broader transcriptional signatures of GC resolution. Module scoring for GC resolution-associated genes (PRDM1, CD93, FOXP3, CCR2, CCL19, SELL) and GC maintenance-associated genes (BCL2L11, TNFRSF9, FAS, CD86, CD83, TNFRSF13B) revealed that mGCs were enriched for resolution-associated programs, whereas cGCs were enriched for maintenance-associated programs (**Figures 6E, F**) (4, 22–24, 38–43). We then examined GC dynamics following Omicron rechallenge. MedLNs were collected 3 days after reinfection (reinfection at 28 dpi, necropsy at 31 dpi). Primary follicle counts were comparable between compartments and time points; however, secondary follicle counts in the medullary compartment were significantly reduced at 31 dpi relative to 21 dpi, whereas cortical secondary follicle counts were maintained (**Figures 6G, H**).

## 4 Discussion

In this study, we identified and characterized a population of GC-like structures arising within the medullary compartment of lung-draining medLNs following SARS-CoV-2 infection in cynomolgus macaques. Our previous study identified the emergence of GC-like B-cell populations within the medullary compartment during convalescence and reinfection (12), but did not define their organization or functional characteristics. Here, we extend those observations by directly comparing medullary and conventional cortical GCs and defining their distinct spatial, cellular, and molecular features. By integrating architectural mapping with spatially resolved transcriptomics, we demonstrated that these LYVE1^+^ sinus-associated medullary GC-like structures, referred to as mGCs, engage core GC-associated programs while adopting a niche-conditioned state. Compared with conventional cGCs, mGCs exhibit altered stromal and T-cell network organization, reduced CXCL12 availability, an increased IgM/IgG ratio, Foxp3^+^ cell enrichment, and decreased persistence of GC-containing follicles following early reinfection.

The nonhuman primate model provided experimental access to lung-draining medLNs, which are rarely accessible in humans, and enabled anatomical and transcriptional analyses that are difficult to achieve in conventional rodent models or human clinical samples (44). Macaque lymph nodes contain well-developed medullary compartments and stromal organization that resemble human lymphoid architecture, providing an ideal platform for interrogating medullary GC biology (45). Consistent with the possibility that GC organization outside conventional cortical follicles is not restricted to nonhuman primates, BCL6^+^ GC activity has also been reported in paracortical/medullary areas of inflamed human lymph nodes (10). This observation suggests that non-cortical GC activity, including within medullary regions, may also occur in humans. Historically, medullary B-cell aggregates have often been regarded as transient, extrafollicular, or structurally incomplete formations, in part because conventional histology alone cannot determine whether such aggregates engage GC-associated programs (11, 46). By combining immunofluorescence with imaging-based spatial transcriptomics, we provided evidence that proliferative medullary clusters activate B-cell activation and cell-cycle-related programs, supporting their interpretation as organized GC-like reactions rather than nonspecific B-cell aggregates.

Our findings indicate that mGCs represent a distinct mode of GC organization shaped by the medullary microenvironment. The shared expression of core GC-associated programs and the consistent detection of organized secondary follicles corroborate that mGCs are structured GC-like reactions. However, mGCs diverge from cGCs across multiple organizational levels. Transcriptionally, cGCs preferentially express selection-associated markers, whereas mGCs show enrichment of stromal, extracellular matrix, and differentiation-associated signatures. Structurally, mGCs lack the polarized FDC and T-cell network topology characteristic of cGCs, suggesting constraints on classical dark zone–light zone compartmentalization (47). At the cellular level, mGCs show reduced CD4^+^ T-cell and GC B-cell densities together with greater plasma cell representation and enrichment of stromal and vascular-associated populations. These observations support the concept that the medulla is not merely a passive conduit for lymphocyte egress or antibody drainage but can provide a specialized niche in which GC programs are deployed in a modified architectural and functional context.

These organizational properties share notable parallels with GC-like structures observed in tertiary lymphoid structures (48). In TLSs, inflammatory stromal remodeling can support GC-like reactions despite incomplete or noncanonical follicular organization (49, 50). Similarly, mGCs retain core GC-associated programs while exhibiting altered stromal architecture and incomplete FDC/T-cell polarization. However, mGCs differ from *de novo* TLSs because they arise within pre-existing lymphoid organs, adjacent to established LYVE1^+^ medullary sinuses, rather than in non-lymphoid tissues. Thus, mGCs may represent either an intermediate organizational state between canonical lymph node GCs and ectopic TLS-associated GCs or a distinct lymph node-resident GC variant shaped by medullary stromal cues. The temporal emergence of mGCs further argues against their interpretation as merely nascent cortical GCs: rare non-cortical proliferative clusters appeared early after infection, whereas organized mGCs became abundant by 21 dpi and were observed across multiple SARS-CoV-2 variants. These findings suggest that medullary GC formation may represent a reproducible feature of antiviral humoral responses in lung-draining lymph nodes.

In addition to altered stromal organization, the cellular composition of mGCs suggests that the medullary compartment may expose GC B cells to a more inflammatory microenvironment than the cortical follicular niche. mGCs contained increased proportions of pDCs and NK cells, which produce inflammatory cytokines and type I interferons that can influence B-cell activation and differentiation (51–53). Consistently, transcriptional profiling showed enrichment of innate immune-associated signatures, including NK-related cytotoxic programs and myeloid/macrophage activation markers (**Supplementary Figure 7**), despite no significant difference in macrophage cell density. Although we did not directly measure local cytokine concentrations, these findings raise the possibility that mGCs are exposed to inflammatory cues such as type I interferons, IFN-γ, or TNF-α, which have been implicated in promoting plasmablast differentiation and antibody-secreting cell responses under inflammatory conditions (54–56). Thus, the differentiation-biased state of mGCs may reflect not only intrinsic B-cell fate programming, but also the combined influence of an inflammation-exposed medullary environment and altered stromal organization.

A central feature of mGCs was their apparent bias toward differentiation rather than prolonged selection. Module scoring revealed that cGCs were enriched for selection-associated genes, whereas mGCs preferentially expressed plasma cell differentiation-associated genes, positioning the two compartments along distinct functional trajectories. This molecular pattern was consistent with greater plasma cell density and a higher IgM/IgG ratio in mGCs. Importantly, these findings should not be interpreted as evidence that mGCs lack GC identity; rather, they suggest that mGCs engage a GC-like program under niche conditions that may be less optimized for sustained iterative selection and more permissive for antibody-secreting cell differentiation. The functional significance of this IgM-skewed output remains unresolved. IgM antibodies can contribute to antiviral immunity through multivalent binding and high-avidity interactions with repetitive viral surface antigens (57, 58), but the same features could also reflect a less mature humoral response characterized by reduced selection stringency, limited affinity maturation, or diminished class-switched antibody output. Distinguishing between these possibilities will require direct functional characterization of mGC-derived antibody repertoires, including somatic hypermutation burden, antigen specificity, epitope recognition, neutralization capacity, and plasma cell longevity.

The CXCL12–CXCR4 axis provides a potential framework for understanding this compartment-specific divergence. GC organization depends on spatially organized chemokine cues that guide B-cell positioning and support cyclic migration between functionally distinct GC zones (1, 47). In our study, the loss of polarized FDC topology in mGCs was accompanied by reduced CXCL12 availability within mGC boundaries. This CXCL12-limited environment coincided with attenuated selection-associated transcriptional signatures, increased differentiation-associated programs, and a higher IgM/IgG ratio. Ex vivo supplementation of macaque primary lymphoid cells with recombinant CXCL12 selectively increased IgG-secreting cell numbers without a corresponding increase in IgM output, providing supportive evidence that CXCL12 availability can influence class-specific antibody output. However, the assay does not establish that CXCL12 alone determines the isotype profile of mGCs *in vivo*. Rather than directly demonstrating defective class-switch recombination, our data suggest that reduced CXCL12 availability may alter the functional organization of GC reactions by influencing B-cell retention, spatial positioning, selection opportunities, and continued participation in GC cycles. Because class-switch recombination can occur before or outside fully developed GC reactions (59), one consequence of this altered organization may be differences in the maintenance, selection, or persistence of class-switched B-cell populations within the medullary niche, potentially contributing to the altered IgM/IgG balance observed in mGCs. Whether the medullary niche also influences plasma cells after GC egress, including their potential local retention through CXCL12-dependent mechanisms in regions adjacent to mGCs, remains to be determined.

mGCs were also distinguished by an altered regulatory T-cell landscape, characterized by FOXP3 enrichment and higher Foxp3^+^ cell density within mGC boundaries. Foxp3-expressing Tfr cells can limit GC magnitude, modulating B-cell selection, and influence GC contraction or cellular egress in specific immune contexts (60, 61). Together with reduced CXCL12 availability, attenuated FDC/T-cell polarization, and differentiation-skewed B-cell programs, these findings indicate that mGCs occupy a regulatory and structural state distinct from cGCs. mGCs also showed enrichment of contraction-associated programs. Because antigen re-exposure can reorganize GC responses (62, 63), we next examined GC dynamics following Omicron rechallenge. Medullary GC-containing follicles were preferentially reduced, whereas cortical GC-containing follicles were maintained. Several interpretations may explain this differential persistence. First, mGCs may represent a transient humoral niche optimized for early antibody-secreting cell output. Second, mGC-derived B cells may redistribute to cortical follicles or other lymphoid niches during recall responses. Third, differences in FDC-mediated antigen retention may contribute to the differential persistence of cGCs and mGCs, as sustained antigen availability supports ongoing GC B-cell selection and affinity maturation (5, 64). The non-polarized FDC organization in mGCs may therefore reflect altered antigen retention or display; testing this possibility will require direct spatial assessment of SARS-CoV-2 antigen. Fourth, the Foxp3^+^ regulatory environment may limit medullary GC persistence upon secondary antigen encounter (40, 41, 60, 61, 65). Because FOXP3 expression alone is insufficient to establish bona fide Tfr identity, these findings should be interpreted as evidence of a Foxp3-enriched regulatory milieu associated with mGC contraction, rather than as proof of Tfr-mediated causality.

Our findings have broader implications for understanding GC heterogeneity and lymph node compartmentalization. Classical models of GC biology have emphasized intra-GC zonation, particularly the dark zone–light zone axis, as the principal framework for understanding B-cell selection and differentiation. Our data extend this framework by showing that anatomical compartmentalization within the lymph node can also shape GC organization and output. In this view, cGCs and mGCs are not simply different locations of the same reaction, but distinct niche-conditioned implementations of the GC program. This compartment-level heterogeneity may be particularly relevant during respiratory viral infection, where antigen drainage, inflammatory cues, lymphatic architecture, and plasma cell egress routes converge within lung-draining lymph nodes. Whether similar medullary GC-like structures arise during other respiratory infections, such as influenza or RSV, remains to be determined.

A critical unresolved question raised by our findings concerns the clonal and developmental relationship between mGCs and cGCs. Recent spatially resolved adaptive immune receptor profiling approaches, including Spatial VDJ and AIR-SPACE, have shown that B-cell clonal architecture can be mapped within native tissue context and that anatomically distinct lymphoid compartments can be linked to clonal diversity and lineage trajectories (66, 67). This framework provides a rationale for asking whether the anatomical segregation of mGCs and cGCs reflects differences not only in location but also in B-cell clonal evolution and functional output. In this context, the LYVE1^+^ sinus-associated positioning and differentiation-biased features of mGCs raise the possibility that anatomical compartmentalization may correspond to a distinct stage or branch of GC B-cell fate, rather than merely reflecting an alternative site of follicular organization. Notably, polyclonal GC responses can export plasma cells across a broad range of clonal and affinity states even under canonical conditions (68), suggesting that the differentiation-biased output observed in mGCs may reflect altered selection or exit dynamics imposed by the medullary niche rather than a departure from GC biology per se. Resolving whether mGC-derived B cells maintain clonal connectivity with cGC lineages, carry distinct somatic hypermutation burdens, or generate antigen-specific antibodies with different functional profiles will require paired spatial transcriptomic and BCR sequencing approaches capable of tracing clonal trajectories across anatomical compartments within the same tissue section.

This study has several limitations. First, the nonhuman primate model inherently limited cohort size and temporal sampling, providing only discrete snapshots of mGC emergence and contraction. In particular, the absence of intermediate sampling between 3 and 21 dpi precluded determination of the precise kinetics of mGC formation. Second, the spatial transcriptomic analysis was based on a targeted 377-gene panel rather than whole-transcriptome profiling, which may incompletely capture GC-associated and stromal programs and limits the discovery of unanticipated transcriptional features. In addition, the focused ELISPOT analysis provided functional support for the specific response examined but did not comprehensively assess functional differences between cGCs and mGCs. Third, although our findings raise important questions regarding the clonal origin and antibody output of mGCs, these issues remain to be directly tested using spatially informed BCR sequencing and functional antibody analyses. Finally, although our findings support the existence of a distinct medullary GC state in SARS-CoV-2-infected macaques, the presence, conservation, and functional relevance of analogous structures in human COVID-19 or other respiratory infections remain unknown.

In summary, our study provides integrative structural and molecular evidence that the lymph node medulla supports a distinct GC-like state during respiratory viral infection. mGCs engage core GC programs but adopt a niche-restricted configuration characterized by altered stromal/T-cell topology, reduced CXCL12 availability, a higher IgM/IgG ratio, Foxp3^+^ regulatory cell enrichment, and lower persistence upon early secondary exposure. These findings highlight anatomical context as a determinant of GC organization and humoral output, and provide a framework for understanding how lymph node microenvironments diversify B-cell responses during infection and possibly vaccination.

## Data Availability

The spatial transcriptomics data presented in this study are publicly available in the NCBI Gene Expression Omnibus (GEO) under accession number GSE346722. The data can be accessed at the following URL: https://www.ncbi.nlm.nih.gov/geo/query/acc.cgi?acc=GSE346722.
