# Supplementary material for: Spatial compartmentalization shapes distinct germinal center states during respiratory viral infection
Source: Front Immunol. 2026 Sep 30;17:1930085. doi: 10.3389/fimmu.2026.1930085 (PMC13661466; doi:10.3389/fimmu.2026.1930085)
Supplement: Supplementary file 1 [file DataSheet1.docx]

Supplementary Material

## 1 Supplementary Figures

**Supplementary Figure 1. Representative immunofluorescence images of medLNs at 21 dpi in SARS-CoV-2 Omicron–infected macaques**

Left: Representative immunofluorescence images of medLNs at 21 dpi stained for CD3 (green), BCL6 (red), and IgD (blue). Right: Single-channel BCL6 signal extracted from the same images, shown in white.

**Supplementary Figure 2. Representative immunofluorescence images of medLNs from SARS-CoV-2–infected macaques at 3 dpi (acute) and 21 dpi (convalescence).**

**(A)** Wuhan strain. Top left: Representative immunofluorescence image of a medLN at 3 dpi (acute phase) stained for CD3 (green), KI67 (red), and IgD (blue). Top right: Representative immunofluorescence image of a medLN at 21 dpi (convalescence), stained for CD3 (green), KI67 (red), and IgD (blue). Bottom left: Single-channel KI67 signal extracted from the 3 dpi image, shown in white. Bottom right: Single-channel KI67 signal extracted from the 21 dpi image, shown in white. **(B)** Delta variant. Top left: Representative immunofluorescence image of a medLN at 3 dpi (acute phase) stained for CD3 (green), KI67 (red), and IgD (blue). Top right: Representative immunofluorescence image of a medLN at 21 dpi (convalescence), stained for CD3 (green), KI67 (red), and IgD (blue). Bottom left: Single-channel KI67 signal extracted from the 3 dpi image, shown in white. Bottom right: Single-channel KI67 signal extracted from the 21 dpi image, shown in white.

**Supplementary Figure 3. Cellular characterization of cortex and medulla germinal centers**

Quantitative comparison of **(A)** CD8⁺ T cells, **(B)** NK cells, **(C)** cDCs, **(D)** pDCs, **(E)** macrophages, **(F)** mast cells, **(G)** fibroblasts, **(H)** vascular endothelial cells, **(I)** lymphatic endothelial cells, **(J)** smooth muscle cells, and **(K)** adipocytes between cGCs and mGCs, presented as counts per mm².

**Supplementary Figure 4. CD35 intensity profiling across cortical and medullary germinal centers**

**(A)** Representative images demonstrating the germinal center (GC) regions of interest (ROIs) used for CD35 intensity measurements. For cortical GCs (cGCs), the measurement axis was defined from the subcapsular sinus (SCS) toward the T–B border. For medullary GCs (mGCs), the axis was defined from the LYVE1⁺ medullary sinuses toward the T–B border. Dashed yellow lines delineate the GC perimeter, and dashed white lines indicate the axis along which CD35 signal intensity was quantified. Corresponding intensity plots are shown to the right. **(B)** CD35 intensity profiles across cortical GCs, with each graph representing one individual GC measured along the SCS-to–T–B border axis. **(C)** CD35 intensity profiles across medullary GCs, with each graph representing one individual GC measured along the LYVE1⁺ sinus–to–T–B border axis.

**Supplementary Figure 5. Spatial distribution of immunoglobulin isotypes in cortical and medullary germinal centers**

**(A)** Immunofluorescence staining of CD3 (green), IgM (red), and IgD (blue) and quantification of IgM expression in cGCs and mGCs. **(B)** Immunofluorescence staining of CD3 (green), IgD (blue), and IgG (orange) quantification of IgG expression in cGCs and mGCs

**Supplementary Figure 6. CXCL12 supports IgG-associated humoral output in lymphoid cell cultures**

**(A)** ELISpot analysis of IgM- and IgG-secreting cells from splenocyte single-cell suspensions cultured with or without CXCL12 (25 ng/mL). The numbers in blue and red represent the counts of IgM- and IgG-secreting cells, respectively. **(B)** Quantification of IgM/IgG ratio from ELISpot assays following CXCL12 treatment. Statistical significance was assessed using the Mann–Whitney U and Wilcoxon signed-rank tests (***p < 0.001; *p < 0.05).

**Supplementary Figure 7.** **Distinct inflammatory module between cGCs and mGCs**

**(A)** Heatmap showing relative expression patterns of cytotoxic and NK cell–associated genes across cGCs and mGCs. mGCs exhibited enrichment of NK/cytotoxicity-related transcripts. **(B)** Heatmap showing relative expression patterns of myeloid/macrophage-associated genes across cGCs and mGCs. mGCs demonstrated increased expression of myeloid/macrophage-associated genes, except for CD14.
